# Carbon Nanocage‐in‐Microcage Structure With Tunable Carbon‐Coated Nickel as a Microwave Absorber With Infrared Stealth Property

**DOI:** 10.1002/advs.202412890

**Published:** 2024-12-17

**Authors:** Zhaoyang Li, Yang Xu, Lihong Wu, Yu Sun, Mingnan Zhang, Zhifeng Dou, Jinchuan Zhao, Yongzhu Yan, Guizhen Wang

**Affiliations:** ^1^ College of Architecture & Civil Engineering Shangqiu Normal University Shangqiu 476000 China; ^2^ Center for Advanced Studies in Precision Instruments School of Material Science and Engineering Hainan University Haikou Hainan 570228 China

**Keywords:** carbon materials, infrared stealth property, microwave absorption capacity, nanocage‐in‐microcage structure

## Abstract

The rational design of microwave absorption (MA) material featuring light weight, wide absorption bandwidth, and infrared stealth property is crucial for military stealth and health protection but remains challenging. Herein, an innovative N‐doped carbon nanocage‐in‐microcage structure with tunable carbon‐coated Ni (NC/Ni(HS)) is reported via a reliable Ni‐catalyzed and Ni‐templated method. The hierarchically hollow structure of nanocage‐in‐microcage composites can optimize the impedance matching and respond to multiple reflections and scattering of incident microwaves and infrared waves. Moreover, the magnetic Ni nanoparticles improve the synergistic interactions between confined heterointerfaces and promote interfacial polarization. Such an ingenious structure endows NC/Ni(HS) with outstanding MA performance and infrared stealth properties. Specifically, NC/Ni(HS)‐10 with an optimal dielectric property, exhibits excellent MA performance. At an ultralow fill loading of 4 wt.%, a wide absorption bandwidth of 6.16 GHz is achieved at a thickness of 2.63 mm, and a strong reflection loss of −63.67 dB is obtained at a thickness of 2.00 mm. In addition, NC/Ni(HS)‐10 shows a low infrared emissivity in the range of 3‒14 µm, which is the key to compatibility with infrared stealth. This work paves the way for the design of advanced MA materials that meet the requirements of multispectral‐compatible stealth.

## Graphical abstract

A microwave absorption material with infrared stealth performance is developed by constructing a N‐doped carbon nanocage‐in‐microcage structure combined with controllable carbon‐coated Ni (NC/Ni(HS)). Their dielectric properties can be effectively tuned by adjusting their etching time. Optimal NC/Ni(HS) exhibits excellent microwave absorption capacity with a reflection loss of −63.67 dB and an effective absorption bandwidth of 6.16 GHz at an ultralow fill loading of 4 wt.%.

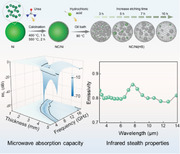

## 1 Introduction

The rapid advancement of wireless communication devices and power transfer technology has caused increasingly serious issues related to electromagnetic radiation and pollution,^[^ ^1^, ^2^, ^3^, ^4^ ^]^ potentially disrupting device operations and harming human body health. Microwave absorption (MA) materials as one of the most effective means to tackle electromagnetic radiation and pollution are highly desired due to they can effectively absorb electromagnetic energy through dielectric or magnetic loss.^[^ ^5^, ^6^ ^]^ To date, a variety of MA materials including metal oxides,^[^ ^7^ ^]^ conducting polymers,^[^ ^8^ ^]^ and ceramics^[^ ^9^ ^]^ have been extensively studied. For practical applications, absorbers are required not only to exhibit strong absorption and wide bandwidth but also to be lightweight. Among various candidates, carbon materials have attracted significant attention due to their high electrical conductivity, large specific surface area, light weight, and robust chemical stability. However, pure carbon materials suffer from certain limitations. Their high electrical conductivity results in the skin effect,^[^ ^10^, ^11^, ^12^ ^]^ leading to impedance mismatch, which increases microwave reflection rather than absorption. Additionally, the single loss mechanism of pure carbon materials typically limits the absorption bandwidth to less than 4 GHz,^[^ ^13^ ^]^ making it challenging for them to achieve strong absorption over a broad frequency range.

Recent advancements in MA materials have demonstrated that incorporating magnetic components, including ferromagnetic metals,^[^ ^14^ ^]^ oxides,^[^ ^15^ ^]^ and alloys^[^ ^16^ ^]^ into carbon materials significantly enhances broadband absorption by improving impedance matching and introducing both dielectric and magnetic losses.^[^ ^17^, ^18^, ^19^ ^]^ Hollow‐structured materials, in particular, show great promise as microwave absorbers, offering lower reflection loss (RL) than bulk counterparts due to their large internal cavities and double‐sided surfaces.^[^ ^20^, ^21^, ^22^ ^]^ The large cavities not only reduce the overall absorber weight but also facilitate multiple reflections and scattering of electromagnetic waves, increasing the effective path length and extending the absorption bandwidth. Additionally, the double‐sided surfaces provide multiple uniform heterogeneous interfaces, which induce strong interfacial polarization effects, thereby improving dielectric loss and further enhancing overall absorption efficiency. Unfortunately, conventional fabrication methods such as high‐temperature pyrolysis, solvothermal methods, and solid template approaches often result in undesirable issues, including nanoparticle aggregation and microstructural collapse.^[^ ^23^, ^24^, ^25^ ^]^ Therefore, achieving precise control over interfacial properties and optimizing the synergy between dielectric and magnetic characteristics are crucial for the development of lightweight, broadband, and high‐performance microwave absorbers.

With the advancement of infrared and radar detection technologies,^[^ ^26^, ^27^, ^28^ ^]^ traditional single‐band stealth materials are no longer sufficient to meet the demands of modern warfare. Consequently, the development of radar/infrared‐compatible stealth materials has become imperative. Here, we address these challenges by employing a novel low‐temperature Ni catalysis and Ni template synergistic strategy to construct a N‐doped carbon nanocage‐in‐microcage structure with tunable carbon‐coated Ni (NC/Ni(HS)). The high crystallinity of graphitized carbon cages facilitates carrier migration to improve conductive loss. Besides, the interior cavity can enhance the impedance matching and induce multiple reflections, while the incorporation of Ni onto the carbon shell via the solid diffusion process promotes interfacial polarization, synergistically improving MA capacity. Furthermore, the hierarchical structure of nanocages confined within a microcage can enhance the MA and infrared stealth performance of NC/Ni(HS) by responding to multiple reflections and scattering of microwaves and infrared waves. Consequently, the NC/Ni(HS) composites exhibit outstanding and controllable MA property. Specifically, at an ultra‐low filling ratio of only 4 wt.%, NC/Ni(HS)‐10 with optimal dielectric property exhibits a minimum RL (RL_min_) of −63.67 dB at 10.16 GHz and an effective absorption bandwidth (EAB) of 6.16 GHz, surpassing the performance of most reported absorbers. Furthermore, NC/Ni(HS)‐10 also shows impressive infrared stealth performance with an infrared emissivity of 0.78. This research offers a paradigm shift in the design and understanding of hollow carbon materials, paving the way for their application in practical multispectral stealth solutions.

## 2 Results and Discussion

### 2.1 Synthesis and Morphology of NC/Ni(HS)

**Figure 1:**
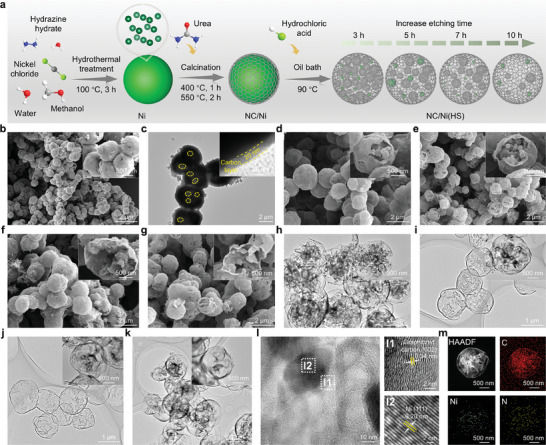
Synthetic and morphological characteristics. a) Schematic illustration of the preparation of NC/Ni(HS). b) SEM image and c) TEM image for NC/Ni. SEM images of d) NC/Ni(HS)‐3, e) NC/Ni(HS)‐5, f) NC/Ni(HS)‐7, and g) NC/Ni(HS)‐10. TEM images of h) Ni‐NG(HS)‐3, i) NC/Ni(HS)‐5, j) NC/Ni(HS)‐7, and k) Ni‐NG(HS)‐10. l) High‐resolution TEM images and m) HAADF‐STEM image and the EDS elemental mappings for NC/Ni(HS)‐10.

**Figure** **1**a presents the synthetic process of NC/Ni(HS) with a nanocage‐in‐microcage structure. Ni microspheres were synthesized by a hydrothermal reaction, which is described in the supporting information. Ni microspheres play several crucial roles in the preparation of NC/Ni(HS): i) as catalysts to catalyze the growth of highly graphitic N‐doped carbon (NC) coating on the Ni surface; ii) as templates to assist in the formation of the hollow structures by etching the Ni core; iii) as Ni sources to incorporate Ni nanoparticles into the NC shells through a solid diffusion process. Following urea pyrolysis, the composites comprising a NC shell and a Ni core (referred to as NC/Ni) were prepared. Finally, the NC/Ni(HS) with well‐constructed hollow structures was obtained by selectively etching the internal Ni core. Scanning electron microscopy (SEM) and transmission electron microscopy (TEM) images display that pristine Ni exhibits a distinct microsphere morphology, characterized by a solid structure and numerous nanosheets on its surface (Figures S1 and S2, Supporting Information). The metallic properties and substantial surface area of Ni microsphere facilitate the decomposition of urea to produce gaseous nitrogen and carbon sources, which are then converted into N‐doped graphitized carbon layers.^[^ ^28^, ^29^, ^30^ ^]^ The NC/Ni intermediate retains the same morphological features as the original Ni (Figure 1b). TEM images and energy dispersive spectroscopy (EDS) elemental mappings of NC/Ni indicate that the Ni microsphere is coated with a uniform carbon layer of ≈20 nm in thickness (Figure 1c; Figure S3, Supporting Information). During the acid etching process, the etched edge gradually becomes highly visible with an extended etching time of 10 h. As expected, the microcage size of samples has no significant change occurring before and after acid etching. Upon removal of the metallic Ni cores by acid, NC/Ni is converted into NC/Ni(HS) (Figure 1d‒g). NC/Ni(HS) samples display nanocage‐in‐microcage structure, which contains a microcage with a wall thickness of ≈20 nm and numerous small nanocages with wall thicknesses ranging from 13 to 25 nm (Figure 1h‒k; Figure S4, Supporting Information). The highly ordered fringes of graphitic carbon are observed in high‐resolution TEM (Figure 1), indicating their high crystallinity.^[^ ^31^ ^]^ In addition, the Ni nanoparticles without obvious aggregation can be seen, suggesting that the good dispersion of Ni nanoparticles is encapsulated in the carbon shell (Figure 1h‒l; Figure S5, Supporting Information). Figure 1 shows a lattice spacing of 0.20 nm, corresponding to the (111) plane of Ni.^[^ ^21^ ^]^ This observation is further corroborated by high‐angle annular dark‐field scanning transmission electron microscopy (HAADF‐STEM) and EDS elemental mapping images (Figure 1m), which shows a uniform distribution of C, Ni, and N elements across the entire NC/Ni(HS).

### 2.2 Structures of NC/Ni(HS)

**Figure 2:**
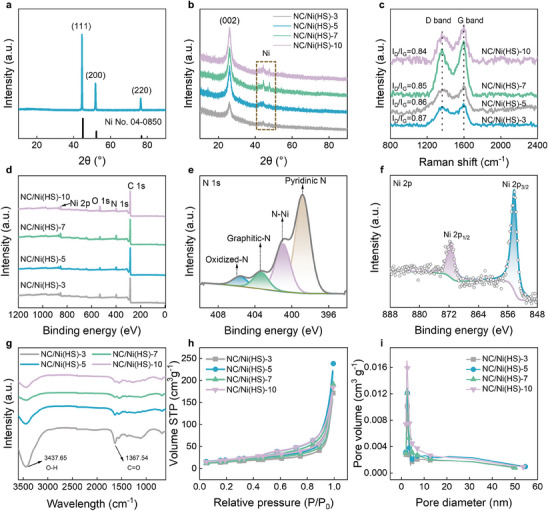
Characterization of as‐obtained NC/Ni(HS)s. a) XRD pattern of NC/Ni. b) XRD patterns, c) Raman spectra, and d) XPS spectra of NC/Ni(HS)s. High‐resolution XPS spectra of e) N 1s core level spectrum and f) Ni 2p core level spectrum of NC/Ni(HS)‐10. g) FTIR spectra, h) N_2_ adsorption‐desorption curves, and i) pore size distribution curves of NC/Ni(HS)s.

The X‐ray diffraction (XRD) pattern of NC/Ni displays typical signals of a face‐centered cubic structure,^[^ ^28^ ^]^ corresponding to the (111), (200), and (220) planes of Ni (**Figure** **2**a). Compared with NC/Ni, the peak of NC/Ni(HS) is relatively weak and broad, indicating that the Ni species are highly dispersed in the form of tiny clusters (Figure 2b). A sharp peak centered at ≈26.2° is observed in the XRD pattern of NC/Ni(HS), which can be attributed to the (002) plane of graphitic carbon,^[^ ^28^ ^]^ indicating the successful removal of the Ni core of NC/Ni(HS). The Raman spectra of NC/Ni(HS)s at 1353 and 1587 cm^−1^ correspond to the D and G bands resulting from the carbon atom lattice defects and carbon atom sp^2^ hybridization (Figure 2c),^[^ ^32^ ^]^ respectively. The *I_D_/I_G_* (intensity ratio) is close to 1, indicating the presence of abundant defects, which may be related to N doping. The surface composition and valence state of NC/Ni(HS) composites were further characterized by X‐ray photoelectron spectroscopy (XPS). The C, N, and Ni elements are observed in Figure 2d, which is consistent with the EDS elements mapping (Figure 1m). The contents of Ni and N in NC/Ni(HS) are 0.87 at% and 6.56 at%, respectively (Table S1, Supporting Information). As displayed in Figure 2e, the binding energies at 398.6, 402.5, and 405.3 eV in the N 1s spectrum correspond to the pyridinic‐N, graphitic‐N, and oxidized‐N, respectively.^[^ ^28^ ^]^ The characteristic peak at 400.7 eV is assigned to Ni─N_x_,^[^ ^33^ ^]^ which means that Ni species are stabilized by N. As for the Ni 2p spectrum (Figure 2f), two main peaks of NC/Ni(HS) are located at 871.6 and 854.3 eV, matching well with the Ni 2p_1/2_ and Ni 2p_3/2_, respectively.^[^ ^21^ ^]^ Fourier transform infrared spectroscopy (FTIR) spectra show two absorption bands at 3437.65 and 1367.54 cm^−1^ (Figure 2g), corresponding to O─H and C═O bonds, respectively, which contribute to the ability of dipole polarization and play an important role in improving the MA capacity. The specific surface area and pore structure of NC/Ni(HS) were analyzed by Brunauer‐Emmett‐Teller (BET) measurement. The obtained NC/Ni(HS) samples show a large specific surface area of 52.29, 68.91, 69.76, 71.54 m^2^ g^−1^ for NC/Ni(HS)‐3, NC/Ni(HS)‐5, NC/Ni(HS)‐7, and NC/Ni(HS)‐10, respectively (Figure 2h). An interconnected mesoporous structure of NC/Ni(HS) is found in the corresponding pore size distributions (Figure 2i), and the macropore of NC/Ni(HS) can be observed from TEM (Figure 1h‒k). Such hierarchical micro‐mesoporous structure of NC/Ni(HS) is expected to be a wide bandwidth microwave absorber by responding to the reflection and scattering of multiple electromagnetic waves.

### 2.3 Efficient MA Performance

**Figure 3:**
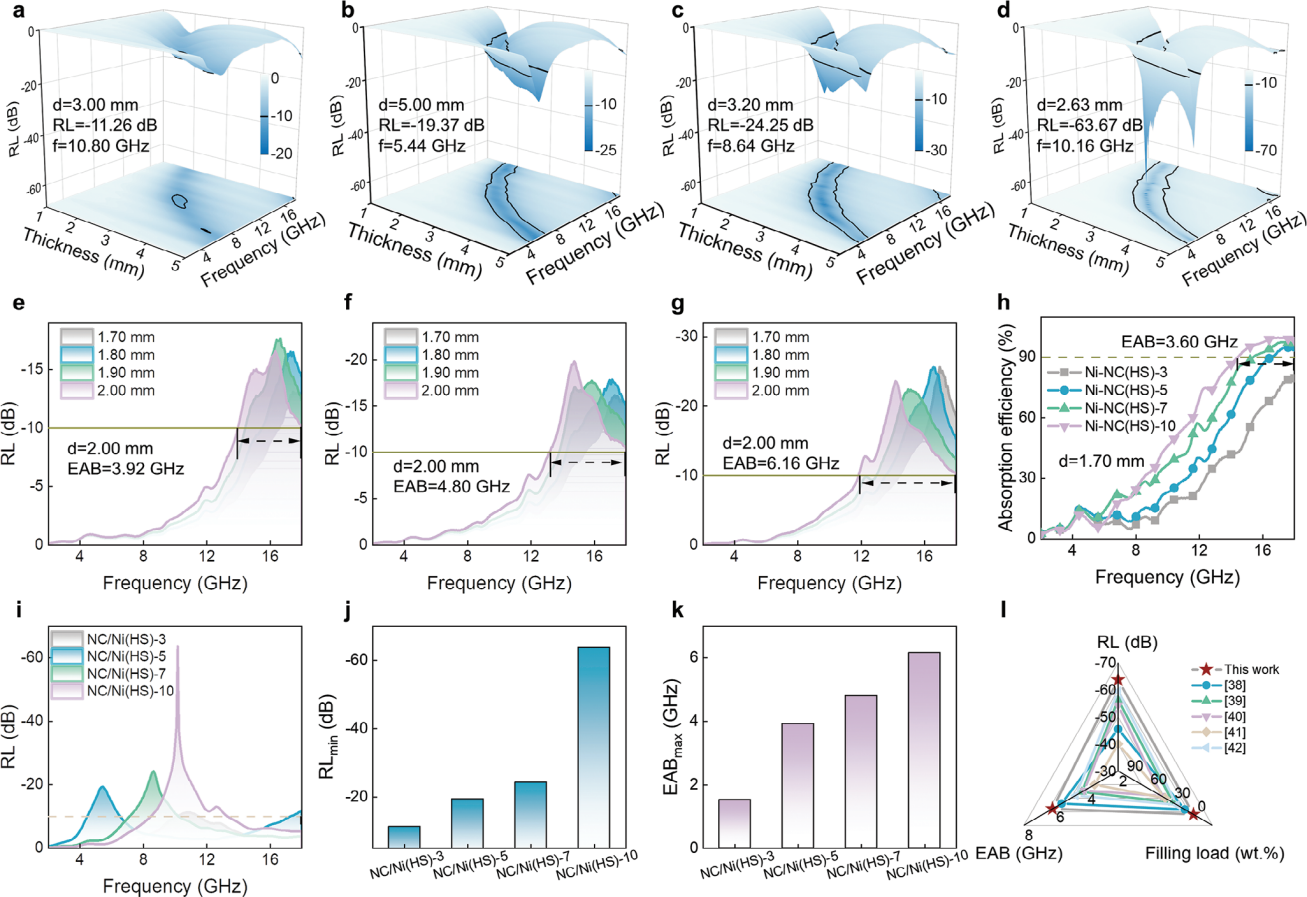
MA properties of NC/Ni(HS)s. 3D diagrams of RL for a) NC/Ni(HS)‐3, b) NC/Ni(HS)‐5, c) NC/Ni(HS)‐7, and d) NC/Ni(HS)−10. RL curves below −10 dB for e) NC/Ni(HS)−5, f) NC/Ni(HS)−7, and g) NC/Ni(HS)−10. h) Comparison of absorption efficiencies of the as‐prepared NC/Ni(HS) samples. i) Optimal RL plots of NC/Ni(HS)−3, NC/Ni(HS)−5, NC/Ni(HS)−7, and NC/Ni(HS)−10. Normalized comparison of j) RL_min_ and k) EAB_max_ values of NC/Ni(HS)−3, NC/Ni(HS)−5, NC/Ni(HS)−7, and NC/Ni(HS)−10. l) Comparison of RL, EAB, and filling ratio values of NC/Ni(HS) with previously reported carbon‐based absorbers.

The MA performance of a material is typically assessed using the RL value (Equations S1 and S2, Supporting Information).^[^ ^34^, ^35^, ^36^ ^]^ An RL value less than −10 dB is considered effective for absorption, indicating that more than 90% of incident electromagnetic waves are absorbed.^[^ ^37^ ^]^ To highlight the advantages of the carbon‐coated Ni nanoparticles anchored hollow porous structure of NC/Ni(HS), the 3D RL plots of the as‐prepared samples were systematically compared. Pure Ni microsphere exhibits no MA performance (no RL≤ −10 dB) with a filling ratio of 4 wt.% and the MA performance does not improve even when coated with NC shells (Figure S6, Supporting Information). Interestingly, with the etching of the Ni core, the MA performance significantly improves. Specifically, NC/Ni(HS)‐3 shows an RL of −11.26 dB at 3.00 mm thickness and an EAB of 1.52 GHz at 3.10 mm thickness (**Figure** **3**a). Notably, the MA capacity enhances markedly with increased etching time, which also reduces the matching thickness. The RL_min_ values for NC/Ni(HS)−5, NC/Ni(HS)−7, and NC/Ni(HS)−10 are −19.37 dB (5.44 GHz), −24.25 dB (8.64 GHz), and −63.67 dB (10.16 GHz) at thicknesses of 5.00, 3.20, and 2.63 nm, respectively (Figure 3b‒d), with the maximum EAB (EAB_max_) values of 3.92, 4.80, and 6.16 GHz at a thin thickness of 2.00 mm (Figure 3e‒g). Compared to NC/Ni(HS)−3, NC/Ni(HS)−10 not only exhibits a reduced matching thickness (from 3.1 to 2.63 mm) but also demonstrates enhanced absorption intensity (from −11.6 to −63.67 dB) and broader EAB (from 1.52 to 6.16 GHz). The RL_min_ and EAB_max_ values of the as‐prepared NC/Ni(HS) samples were systematically compared (Figure 3h‒k), with NC/Ni(HS)−10 showing significant advantages at thin absorption thicknesses. Furthermore, the absorption peak can be tuned from the C‐band (4−8 GHz) to the Ku‐band (12−18 GHz) by tailoring the aching etching time (Figure 3k). As illustrated in Figure 3l, NC/Ni(HS)‐10 outperforms most previously reported carbon‐based absorbers regarding RL_min_ and EAB_max_ features. In particular, such excellent MA performance is achieved at an ultra‐low filling ratio of only 4 wt.%, which greatly surpasses the reported filling ratio values.^[^ ^38^, ^39^, ^40^, ^41^, ^42^ ^]^

**Figure 4:**
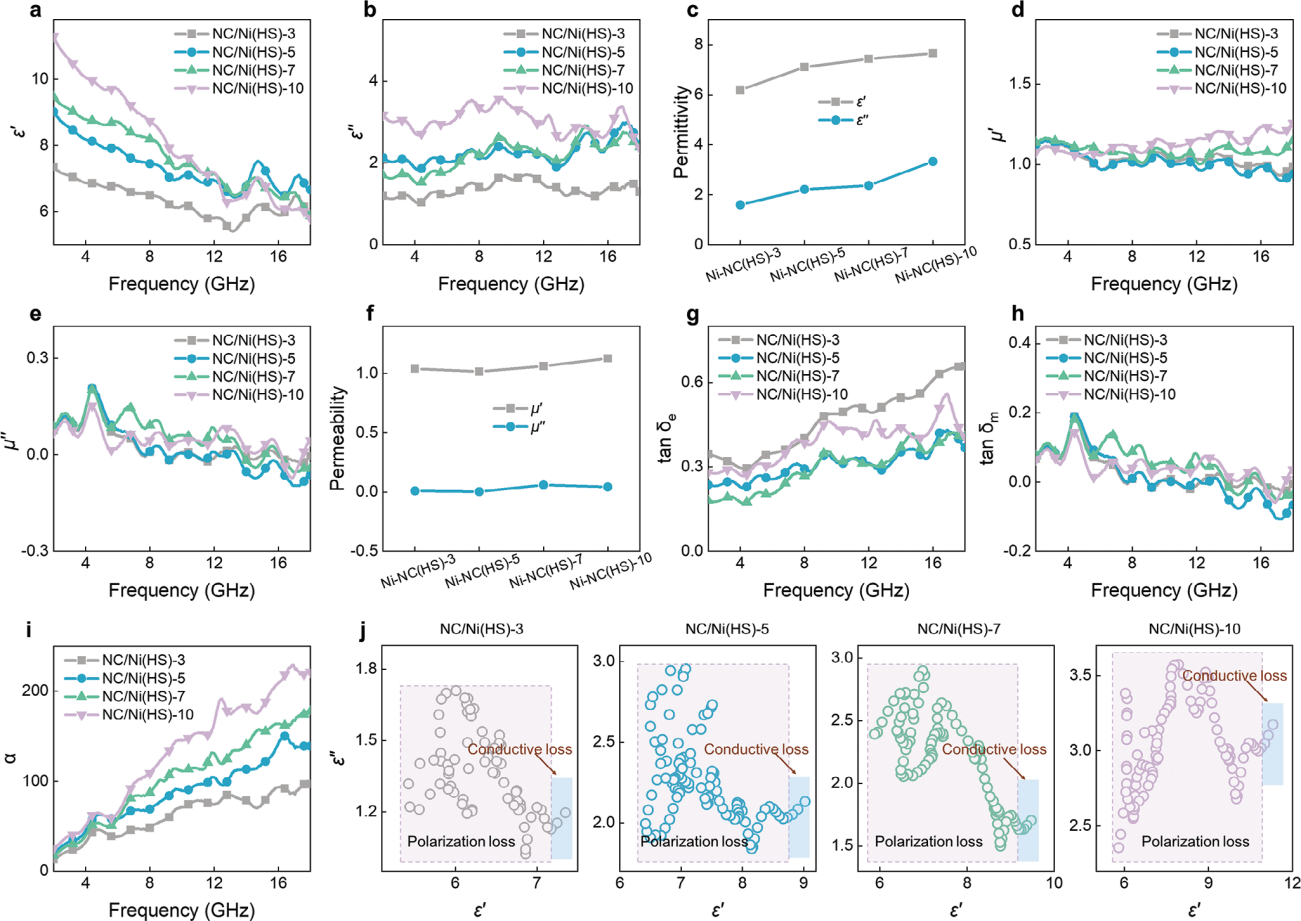
Electromagnetic parameters and attenuation ability of NC/Ni(HS)s. Frequency‐dependent a) *ε*′ and b) *ε*′′ curves. c) Comparison of *ε*′ and *ε*′′ at the frequency of 10.16 GHz. Frequency‐dependent d) *μ*′ and e) *μ*′′ curves. f) Comparison of *μ*′ and *μ*′′ at the frequency of 10.16 GHz. g) tan δ_e_, h) tan δ_m_, i) α, and j) Cole‐Cole curves.

The absorption properties of microwave absorbers are related to electromagnetic parameters, coating thickness, and matching frequency, which are determined by their composition, structure, morphology, and size.^[^ ^10^ ^]^ In this work, the exceptional MA performance of NC/Ni(HS) can be attributed to the following factors. The enhancement in MA originates from the efficient combination of hollow structures of magnetic Ni and NC. RL can be improved when the dielectric and magnetic contributions are matched, based on input impedance requirements. The real (*ε*′) and imaginary (*ε*ʺ) parts of complex permittivity refer to the ability to store and dissipate electrical energy, respectively. Similarly, the real (*μ*′) and imaginary (*μ*ʺ) parts of complex permeability represent the capability of magnetic energy storage and dissipation, respectively. To further investigate the associated mechanism for the diverse MA capacity of obtained samples, the permittivity (*ε*′ and *ε*″) and permeability (*μ*′ and *μ*″) were plotted (**Figure** **4a‒d**; Figures S7 and S8, Supporting Information). Ni and NC/Ni display almost constant *ε*′ values of 3.0 and 2.6, respectively, while their *ε*″ values are close to 0, implying the ability of Ni and NC/Ni to absorb electromagnetic energy is limited. According to previous reports, introducing hollow structures is an effective strategy to improve impedance matching and dielectric dissipation capacity. As expected, the *ε*′ and *ε*″ values rapidly increase with the prolonged acid etching time (Figure 4a,b). In the frequency range of 2.00‒18.00 GHz, the *ε*′ values of NC/Ni(HS)−3, NC/Ni(HS)−5, NC/Ni(HS)−7, and NC/Ni(HS)−10 vary from 7.3 to 5.9, from 9.0 to 6.7, from 9.5 to 5.9, and from 11.3 to 5.8, respectively. Simultaneously, the *ε*″ values of NC/Ni(HS)‐3, NC/Ni(HS)−5, NC/Ni(HS)−7, and NC/Ni(HS)−10 vary from 1.7 to 1.2, from 2.4 to 1.8, 2.9 from 1.5, and from 3.6 to 2.4, respectively. According to the free electron theory (*ε*ʺ ≈ *𝜎*/2*𝜋ε_0_f*, where *𝜎* and *ε_0_* represent the electrical conductivity and free space impedance, respectively), *ε*ʺ is positively correlated with *𝜎*, and a large *𝜎* facilitates the migration and jumping of electrons, thereby increasing the conduction loss.^[^ ^12^, ^43^ ^]^ Further analysis of the complex permittivity of NC/Ni(HS)−3, NC/Ni(HS)−5, NC/Ni(HS)−7, and NC/Ni(HS)−10 at 10.16 GHz shows that NC/Ni(HS)−10 exhibits the highest *ε*′ and *ε*″, corresponding to the position of the strongest absorption peak of NC/Ni(HS)−10. Figure 4d‒f and Figure S8 (Supporting Information) display the complex permeability curves of obtained samples. *μ*′ increases gradually with increasing etching time, likely due to the uniform distribution of Ni in the carbon layer. Additionally, several natural resonance peaks are found in the *μ*″ curves, which may be derived from loss and magnetic coupling effect.^[^ ^44^ ^]^ NC/Ni(HS)‐10 has the highest *μ*′ and similar *μ*″ value at 10.16 GHz, which favors improving impedance matching and MA absorption performance.

The dielectric tangent loss (tan 𝛿_e_ = *ε*′/*ε*″) and magnetic tangent loss (tan 𝛿_m_ = (*μ*′/*μ*″) are typically applied to assess the dielectric loss and magnetic loss ability of absorbers, respectively. Figure 4g,h, and Figure S9 (Supporting Information) indicate that the NC/Ni(HS)−10 possesses moderate dielectric and magnetic attenuation capabilities. All the tan 𝛿_e_ values vary between 0.18 to 0.66, while tan 𝛿_m_ values are less than 0.2. These results suggest that the attenuation capability of NC/Ni(HS) composites for microwave mainly relies on the dielectric loss. The attenuation constant (*α*) is a crucial factor for the MA property of absorbers, determining the loss ability of incident microwaves (Equation S3, Supporting Information).^[^ ^6^ ^]^ As shown in Figure 4i and Figure S10 (Supporting Information), the *α* values of NC/Ni(HS) composites increase with prolonged etching time and frequency. The behavior of dielectric loss can be described by Cole‐Cole curves (Equations S4 and S5, Supporting Information).^[^ ^9^, ^43^ ^]^ Slender tails and multiple semicircles observed in the curves of NC/Ni(HS) samples imply the occurrence of conductive loss and polarization relaxation processes (such as interfacial polarization and dipole polarization) (Figure 4j). This may be attributed to the presence of abundant heterogeneous interfaces and defects. Due to the differences in the dielectric properties at different interfaces, charges accumulate at the Ni/NC and air/NC interfaces, resulting in strong interfacial polarization. The dipole polarization is related to vacancy defects in the graphitic region and the doping of N atoms. These enriched defects can act as dipole active sites, disrupt the lattice periodicity, tune the electric field, and alter the electrical transmission path, thus enhancing the dielectric loss.

**Figure 5:**
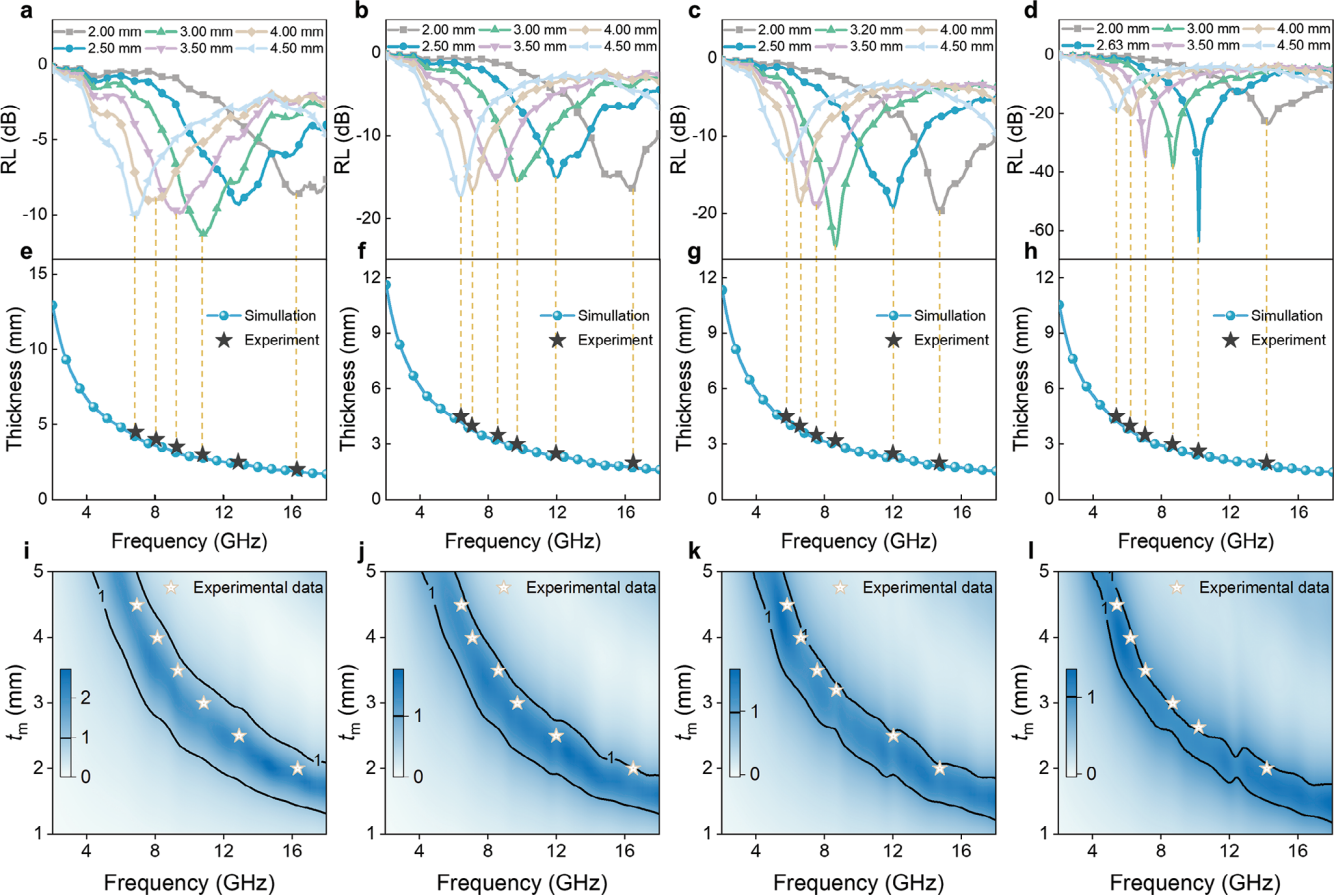
MA performance, dissipation loss mechanism, and impedance matching. The RL values at various thicknesses for a) NC/Ni(HS)−3, b) NC/Ni(HS)−5, c) NC/Ni(HS)−7, and d) NC/Ni(HS)−10. Theoretical matching thickness curves of e) NC/Ni(HS)−3, f) NC/Ni(HS)−5, g) NC/Ni(HS)−7, and h) NC/Ni(HS)−10. 2D projections of the *Z* values (black lines representing the *Z* value of 1) correlating the thickness and the frequencies for i) NC/Ni(HS)−3, j) NC/Ni(HS)−5, k) NC/Ni(HS)−7, and l) NC/Ni(HS)‐10.

The absorption frequency of microwave absorbers can be adjusted by customizing their matching thickness. **Figure** 5a‒d illustrates the thickness‐dependent RL curves, demonstrating that as the matching thickness of the absorber increases, the absorption peak shifts toward a lower frequency. The quarter‐wavelength (*λ*/4) theory can be used to explain the relationship between the absorption peak and matching thickness (Equation 1).^[^ ^45^ ^]^

(1) $$t_{m}=\frac{n\lambda }{4}=\frac{nc}{4f_{m}\sqrt{\left|\varepsilon_{r}\mu_{r}\right|}}\left(n=1,3,5\text{…}.\right)$$

Here, *t*_m_ is the matching thickness, while *λ*, *c*, and *f*_m_ stand for wavelength, the speed of light, and matching frequency, respectively. Clearly, the thickness is inversely proportional to frequency. According to the *λ*/4 theory,^[^ ^46^ ^]^ interference cancellation can occur when the phase difference between the reflected wave at the air‐absorber interface and the reflected wave at the absorber‐conductive background interface is 180, resulting in enhanced absorption intensity. Moreover, the black pentagrams (experimental data) slightly deviate from the *λ*/4 matching model (Figure 5e‒h), likely due to the presence of multiple microwave loss mechanisms in the system. It is well known that an ideal microwave absorber not only requires large attenuation capability but also needs to meet impedance matching requirements. The impedance matching (*Z* = |*Z*_in_/*Z*_0_|) describes the ability of incident microwaves to penetrate the absorber instead of being reflected back into the air.^[^ ^45^ ^]^ When the *Z* value is close to 1, microwaves can easily penetrate the material, which is a prerequisite for obtaining good MA performance.^[^ ^9^ ^]^ The frequency‐dependence *Z* values at various thicknesses are plotted in Figure 5i‐l. Compared to NC/Ni(HS)−3, the other samples exhibit good impedance matching, with NC/Ni(HS)−10 showing the best effect.

### 2.4 RCS Simulation

**Figure 6:**
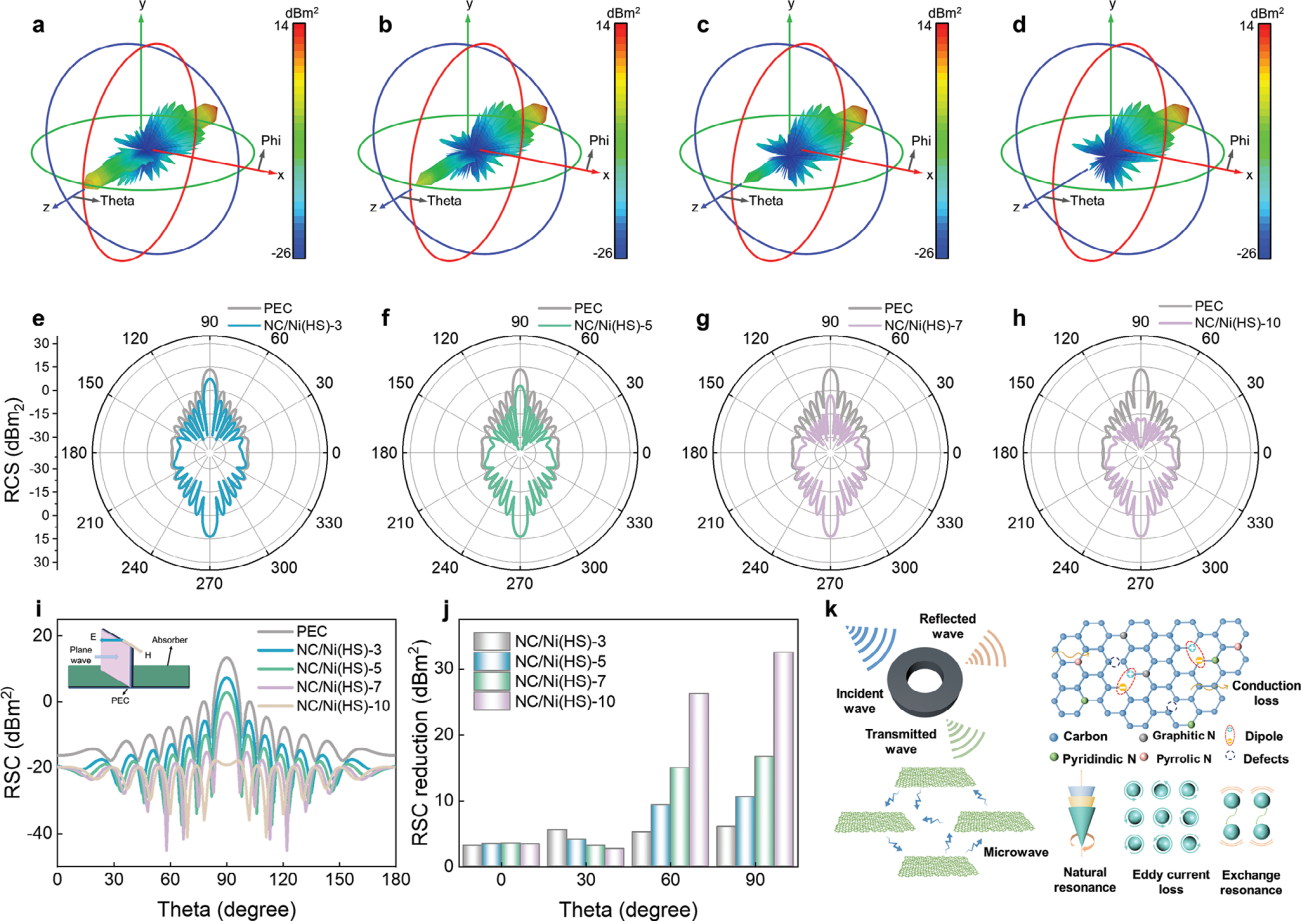
CST simulation results. 3D RCS diagram for the PEC substrate covered with a) NC/Ni(HS)‐3, b) NC/Ni(HS)‐5, c) NC/Ni(HS)‐7, and d) NC/Ni(HS)‐10. RCS in polar coordinate system for e) NC/Ni(HS)‐3, f) NC/Ni(HS)‐5, g) NC/Ni(HS)‐7, and h) NC/Ni(HS)‐10. i) Simulated RCS values of samples in the Cartesian coordinate RSC values from 0° to 180° (inset: the model of CST simulation). j) Comparison of the RCS reduction values. k) MA mechanism of NC/Ni(HS) composites.

The microwave stealth property of absorbers is critical for effective anti‐radar detection. Radar cross section (RCS) serves as a vital metric for evaluating the MA performance of materials under actual far‐field conditions.^[^ ^47^, ^48^, ^49^ ^]^ **Figure** **6**a‒d and Figure S11 (Supporting Information) present the 3D radar plane wave scattering signals of the perfect conductive plate (PEC) and the PEC plate covered by NC/Ni(HS)s. Compared to the PEC plate, the reflection signals of all NC/Ni(HS) samples are effectively suppressed, with NC/Ni(HS)‐10 exhibiting the weakest reflection intensity. This implies that NC/Ni(HS)‐10 absorbs more microwave energy. Figures 6e‒h further demonstrate that NC/Ni(HS)‐10 exhibits the smallest RCS, aligning well with the excellent MA performance of the obtained NC/Ni(HS) samples. Figure 6i shows 2D fluctuation plots of RCS values with a detection degree range from 0° to 180°. It can be observed that the proportion of reflected microwaves is highest when incident vertically on the model plane, gradually decreasing from the detection degree of 90° to 0° and 180°. The PEC plate shows the maximum RCS signal of 13.4 dBm^2^, while the NC/Ni(HS)‐10 coated plate exhibits a minimum RCS value (below −17.8 dBm^2^ at all detection degrees) at 10.16 GHz in the X‐band. RCS reduction (the RCS values of PEC minus that of samples) was further employed to evaluate the microwave energy dissipation capacity of as‐prepared samples, as depicted in Figure 6j. At *θ* = 90°, the RCS reduction values of NC/Ni(HS)‐3, NC/Ni(HS)‐5, NC/Ni(HS)‐7, and NC/Ni(HS)‐10 reach maximums of 6.04, 10.59, 16.72 and 32.48 dBm^2^, respectively, then gradually decrease as the angle decrease. These findings indicate that the microwave energy loss is influenced not only by the intrinsic electromagnetic response properties but also by the detection angles. Consequently, NC/Ni(HS)‐10 proves suitable for practical applications in complex radar detection environment.

With the above analyses, the excellent MA performance of NC/Ni(HS)‐10 can be mainly ascribed to its Ni‐incorporated NC nanocages‐in‐microcage structure. Here are the key factors contributing to its superior microwave absorption property (Figure 6k):

i) Enhanced conductive loss. The cavities provide a large surface area and graphitized carbon enhances conductivity. This facilitates the formation of numerous conductive networks and pathways for charge transfer, leading to improved conductive loss.

ii) Multiple reflections and absorption. The hollow structure facilitates multiple reflections and absorption of incident microwaves, increasing the chances of energy dissipation within the material.

iii) Polarization relaxation. The rich NC and heterogeneous interfaces between carbon and air, and carbon and Ni, contribute to enhanced dipole polarization and interfacial polarization, respectively.

iv) Synergistic effects. The interaction between carbon and Ni can distort electron distribution, changing the polarity of lattice points. This interaction, coupled with multiple polar units, further enhances the dielectric loss capacity. By achieving good impedance matching, utilizing multiple polar units, and leveraging the synergistic effect of various loss mechanisms, NC/Ni(HS)‐10 exhibits excellent microwave absorption capacity.

### 2.5 Infrared Stealth Property

**Figure 7:**
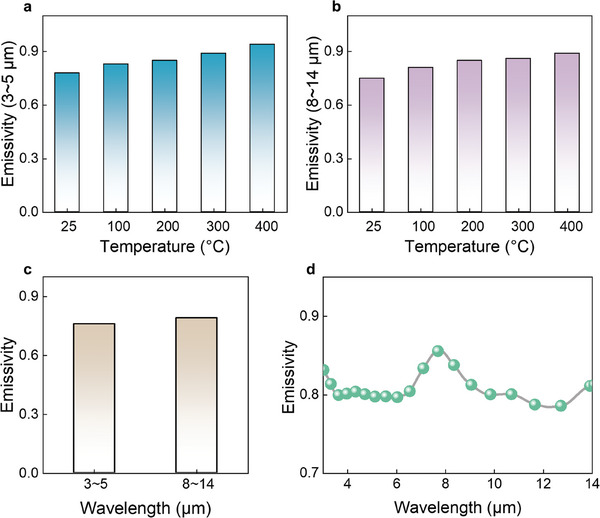
Infrared stealth performance. a) Mid‐ and b) long‐wavelength infrared emissivity at various temperatures. c) Comparison of infrared emissivity. d) Wavelength dependence of infrared emissivity.

Low infrared emissivity is important for achieving infrared stealth.^[^ ^19^, ^50^ ^]^ To demonstrate the infrared stealth property of NC/Ni(HS)‐10, the infrared emissivity of NC/Ni(HS)‐10 in the range of 25 to 400 °C was analyzed using an FTIR spectrometer and blackbody radiation. Infrared radiation characteristics can be reduced by adjusting the nodal emissivity without changing the surface temperature of the material. In the atmospheric environment, infrared radiation of different wavelengths will be attenuated by different degrees. The main operating bands of infrared detectors are in the relatively transparent atmospheric window regions of 3‒5 and 8‒14 µm.^[^ ^18^ ^]^ Due to its excellent MA performance, NC/Ni(HS)‐10 was chosen for the detailed analysis. **Figure** **7**a,b shows the variation of infrared emissivity of NC/Ni(HS)‐10 with temperature in the 3‒5 and 8‒14 µm bands, respectively. The infrared emissivity of NC/Ni(HS)‐10 increases slightly from room temperature to 400 °C. The average emissivity is only 0.76 (3‒5 µm) and 0.79 (8‒14 µm) (Figure 7c). Figure 7d shows a more detailed infrared emissivity curve as a function of wavelength. The larger specific surface area of NC/Ni(HS)‐10 facilitates internal refraction and absorption of infrared light, which attenuates the infrared radiation energy emitted by the target, resulting in lower infrared emissivity. The special hollow channel structure enhances thermal insulation performance, reducing the infrared radiation energy density of the sample. The smooth surface and uniform particle size of the NC/Ni(HS)‐10 samples contribute to lowering emissivity, as there is a positive correlation between surface roughness and particle size uniformity and emissivity. The air space between the shells in the hollow structure acts as a thermal insulator, further decreasing the infrared radiation energy density.

## 3 Conclusion

In summary, NC/Ni(HS) was successfully synthesized via a simple Ni‐catalyzed and Ni‐templated approach. Compared to Ni and NC/Ni, NC/Ni(HS) exhibits significantly improved MA performance under a filling ratio of merely 4 wt.%, delivering a wide absorption bandwidth (6.16 GHz at only 2.00 mm) and strong absorption intensity (−63.67 dB at 2.63 mm). The remarkable MA capacity may be related to its unique Ni‐incorporated NC nanocage‐microcage structure. The high crystallinity and a large number of defects of NC/Ni(HS) allow for efficient electron transfer and jump and provide more polarization sites, helping to improve dielectric loss capacity. Specifically, by changing the etching time of Ni, the hollow structure of NC/Ni(HS) was efficiently tuned, contributing to the accurate control of the impedance matching and conductive loss. In addition, NC/Ni(HS) also possesses a low infrared emissivity, ensuring infrared stealth. Our work provides a novel inspiration for the design and fabrication of advanced microwave absorbers with infrared stealth in aerospace, aircraft, and other fields.

## Conflict of Interest

The authors declare no conflict of interest.

## Supporting information



Supporting Information

## Data Availability

The data that support the findings of this study are available from the corresponding author upon reasonable request
