# Supplementary material for: Carbon Nanocage‐in‐Microcage Structure With Tunable Carbon‐Coated Nickel as a Microwave Absorber With Infrared Stealth Property
Source: Adv Sci (Weinh). 2024 Dec 17;12(6):2412890. doi: 10.1002/advs.202412890 (PMC11809319; doi:10.1002/advs.202412890)
Supplement: Supplementary file 1 — Supporting Information [file ADVS-12-2412890-s001.docx]

Supporting Information

**Carbon** **Nanocage-in-Microcage Structure with Tunable** **Carbon-Coated Nickel as a Microwave Absorber with Infrared Stealth Property**

Zhaoyang Li*, Yang Xu, Lihong Wu*, Yu Sun, Mingnan Zhang, Zhifeng Dou, Jinchuan Zhao, Yongzhu Yan, and Guizhen Wang*

Z. Li, Y. Sun, G. Wang

College of Architecture & Civil Engineering, Shangqiu Normal University, Shangqiu 476000, China.

E-mail: lizhaoyang@sqnu.edu.cn (Z. Li)

Email: wangguizhen0@hotmail.com (G. Wang)

Y. Xu, L. Wu, M. Zhang, Z. Dou, J. Zhao, Y. Yan, G. Wang

State Key Laboratory of Marine Resource Utilization in South China Sea, Center for Advanced Studies in Precision Instruments, Hainan University, Haikou, Hainan 570228, China

Email: wulihong@hainanu.edu.cn (L. Wu)

Email: wangguizhen0@hotmail.com (G. Wang)

**Synthesis of Ni Microspheres**

At room temperature, 500 mg of Nickel chloride hexahydrate (NiCl_2_·6H_2_O, AR; Aladdin) was dissolved in a mixed solution containing 5 mL of methanol (CH_3_OH, 99.5%; Xilong Chemical) and 15 mL of deionized water under magnetic stirring. Then, 800 μL of hydrazine hydrate (N_2_H_4_·H_2_O, 80%; Aladdin) was added dropwise to the above solution under vigorous magnetic stirring to form a lavender solution. The mixed solution was maintained at 100 °C for 3 h (100 mL autoclave). The products were obtained by washing (deionized water and ethanol several times) and drying under vacuum (70 °C for 12 h).

**Synthesis of N-doped Carbon/Ni (NC/Ni)**

200 mg Ni microspheres and 500 mg urea (CH_4_N_2_O, AR; Macklin) were placed in the same ceramic boat, in which urea was positioned at the inlet of a tubular furnace under an Ar atmosphere. A two-stage annealing process was employed to treat the precursor. Firstly, the temperature was increased to 400 °C at a rate of 2 °C min^−1^ and maintained for 1 h. The Ni can facilitate the decomposition of urea to produce gaseous N and C sources, which are then converted into N-doped graphitized C layers. Then, the temperature was further raised to 550 °C at a rate of 2 °C min^−1^ and held for 2 h. In this progress, the Ni microsphere is gradually eroded and finally transformed into Ni nanoparticles. At the same time, C and N compounds will cover the surface of Ni nanoparticles and be catalyzed to form an N-doped carbon coating. The resulting product was designated as NC/Ni.

**Synthesis of Hollow Carbon-Coated Ni/Carbon Nanocage-in-Microcage Composites (NC/Ni(HS))**

The obtained NC/Ni was immersed in 1 M HCl (HCl, 37%; Aladdin) and kept at 90 °C in an oil bath separately for 3, 5, 7, and 10 h to remove Ni and other unstable substances. The resulting black suspension was filtered, washed (deionized water and ethanol several times), and dried at 60 °C for 12 h. The final products were named as NC/Ni(HS)-3, NC/Ni(HS)-5, NC/Ni(HS)-7, and NC/Ni(HS)-10

**Apparatus**

The phase composition and crystal structure were recorded by X-ray diffraction (XRD; Smart Lab Ⅱ). X-ray photoelectron spectroscopy (XPS) data were characterized by AXIS SUPRA. Scanning electron microscopy (SEM), and energy dispersive spectroscopy (EDS) mappings were performed by using the Thermo Scientific Verios G4 UC microscope. Transmission electron microscopy (TEM), and EDS mapping of samples were collected by using the transmission electron microscopy system (Thermo Scientific, Talos F200X G2). Fourier transform infrared spectroscopy (FTIR) was investigated by a Tensor 27 spectrometer. Raman spectra were acquired using inVia Reflex (Renishaw) with a 514 nm laser. The specific surface area and pore size distribution were determined by N_2_ adsorption-desorption isotherms (Micromeritics ASAP2460).

**Infrared Emissivity Test**

NC/Ni(HS)-10 was pressed into solid circular pieces with a thickness of about 2 mm. The emissivity property of prepared samples at the wavelengths of 3–5 and 8–12 μm was obtained using FTIR (Nicolet is50+Nicolet continum) with testing temperatures ranging from room temperature to 400 °C.

**Microwave Absorption (MA) Performance Test**

To measure the MA properties of as-synthesized NC/Ni(HS) composites, the coaxial transmission line method was applied and a vector network analyzer (Ceyear, 3672B-S) was used to obtain the EM parameters. The obtained NC/Ni(HS) were mixed with paraffin at a mass ratio of 4 wt.%, followed by shaping coaxial rings with an outer diameter of 7.00 mm and an inner diameter of 3.04 mm (2.00 mm in thickness).

**Simulation**

A radar cross-section (RCS) simulation was performed to evaluate the actual far-field condition MA capacity in real situations, which is simulated by CST Studio Suite 2020. The frequency of 10.16 GHz is selected. According to the widely accepted metal back model, the simulation model of the specimens was established as a square (200 mm × 200 mm) with dual layers. In detail, the bottom set as 0.5 mm is the perfect conductive layer (PEC), and the upper set as 2.63 mm signifies the absorbing layer for NC/Ni(HS). The PEC and the absorbing model plate were placed on the X–O–Y plane and linear polarized plane electromagnetic waves incident from the positive direction of the Z-axis to the negative direction of the Z-axis. Simultaneously, the direction of electric polarization propagation is along the X-axis. Open (add space) boundary conditions were employed in the x, y, and z directions. Generally, the scattering directions of RCS value can be determined by theta and phi in spherical coordinates, which can be described below:

$\sigma=10log(\left( 4\pi S/\lambda^{2} \right){\left| E_{S}/E_{i} \right|)}^{2}$

Here, S, λ, Es, and Ei are the area of the target object simulation model, the wavelength of electromagnetic wave, the electric field intensity of scattered wave, and the incident wave, respectively.

**Equations**

$Z_{in}=Z_{0}\sqrt{{\mu_{r}}/{\varepsilon_{r}}}\tanh[j({2\pi fd}/{c)\sqrt{\mu_{r}\varepsilon_{r}}}]$ (S1)

$RL=20\lg\left| {(Z_{in}-Z_{0})}/{(Z_{in}+Z_{0})} \right|$ (S2)

Where Z_in_ is the input impedance of the absorber, *Z*_0_ is the impedance of free space, $\mu_{r}$ is the relative complex permeability ($\mu_{r}=\mu'-i\mu"$), $\varepsilon_{r}$ is the complex permittivity ($\varepsilon_{r}=\varepsilon'-i\varepsilon"$), *d* is the thickness of absorber, *c* and *f* represent velocity and frequency of light, respectively.

The attenuation constant (α) represents the attenuation capacity of EM wave, which is usually expressed as follows:

$\alpha=\frac{\sqrt{2}\pi f}{c}\times\sqrt{\left( \mu"\varepsilon"-\mu'\varepsilon' \right)+\sqrt{\left( \mu"\varepsilon"-\mu'\varepsilon' \right)^{2}+\left( \mu"\varepsilon'+\mu'\varepsilon" \right)^{2}}}$ (S3)

In general, the dielectric loss can be described by Debye’s theory:

$\varepsilon'=\varepsilon_{\infty}+\frac{\varepsilon_{s}-\varepsilon_{\infty}}{1+\left( \omega\tau_{0} \right)^{2}}$ (S4)

$\varepsilon"=\frac{\omega\tau_{0}\left( \varepsilon_{s}-\varepsilon_{\infty} \right)}{1+\left( \omega\tau_{0} \right)^{2}}+\frac{\sigma}{\omega\varepsilon_{0}}$ (S5)

where, $\varepsilon_{\infty}$ is the relative dielectric permittivity in the high-frequency limit, *𝜏* is the polarization relaxation time, 𝜔 is the angular frequency, $\varepsilon_{s}$ is the static permittivity, $\varepsilon_{0}$ is the dielectric constant of vacuum, *σ* is the electrical conductivity.

Table S1 Component analysis of the NC/Ni(HS) samples.

| Samples | C (at %) | N (at %) | O (at %) | Ni (at %) |
| --- | --- | --- | --- | --- |
| NC/Ni(HS)-3 | 87.07 | 6.61 | 4.90 | 1.43 |
| NC/Ni(HS)-5 | 86.73 | 8.52 | 3.42 | 1.33 |
| NC/Ni(HS)-7 | 91.07 | 5.17 | 2.80 | 0.96 |
| NC/Ni(HS)-10 | 88.05 | 6.56 | 4.52 | 0.87 |


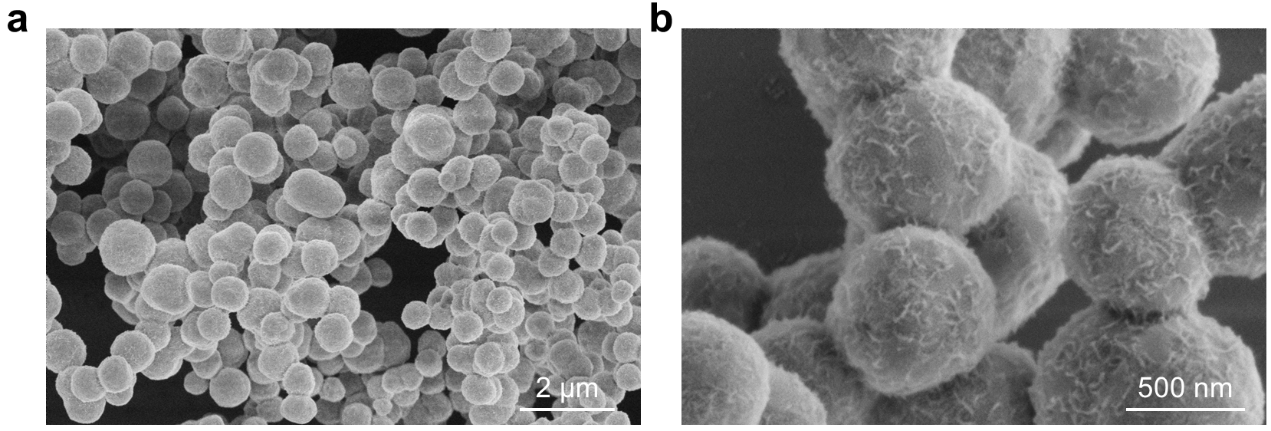


**Figure S1.** SEM images of Ni microsphere.


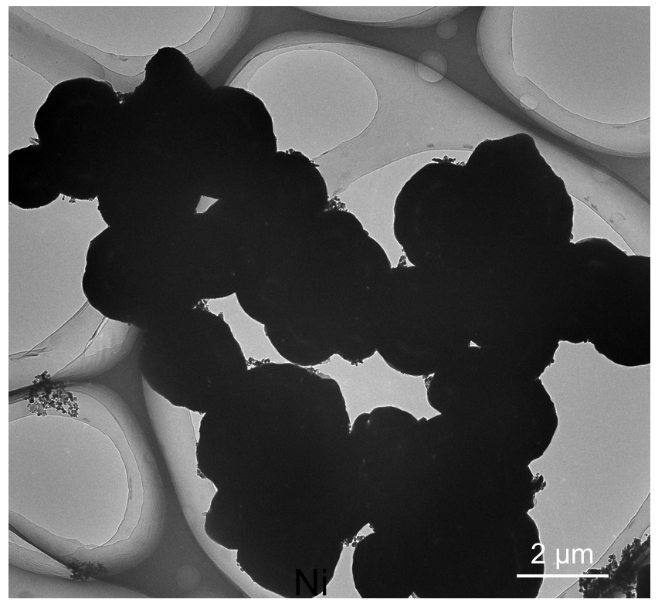


**Figure S2.** TEM image of Ni microsphere.


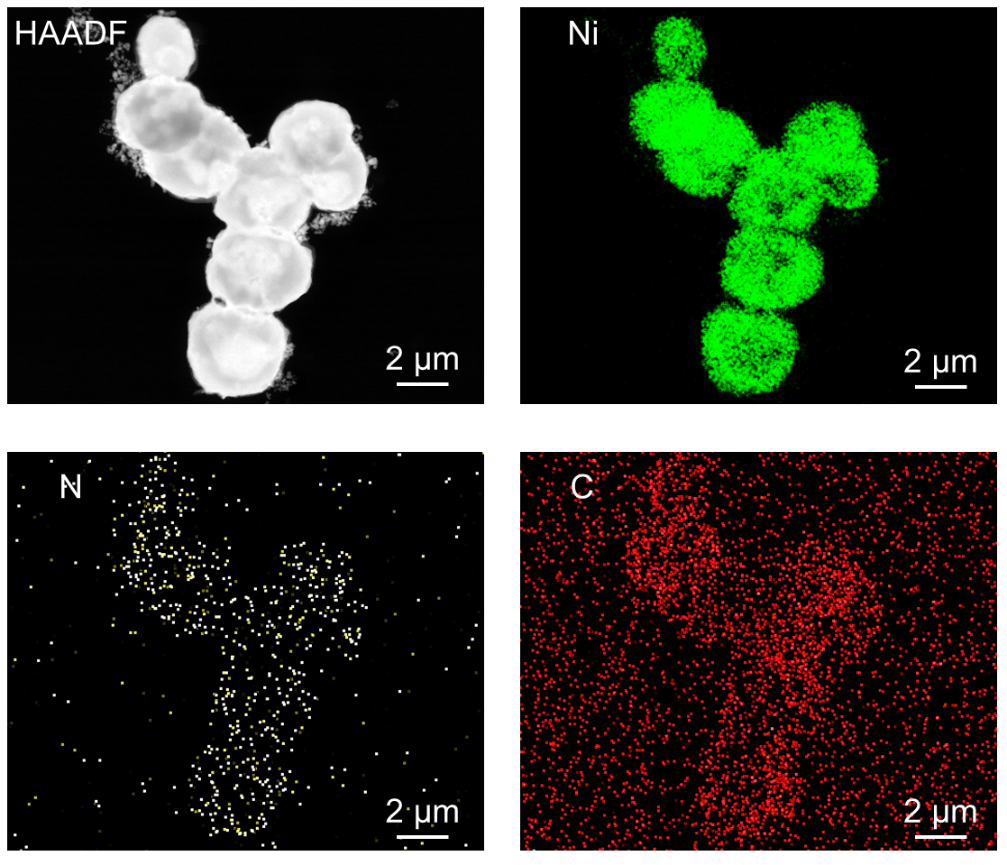


**Figure S3.** The HAADF image and EDS elemental mappings of NC/Ni.


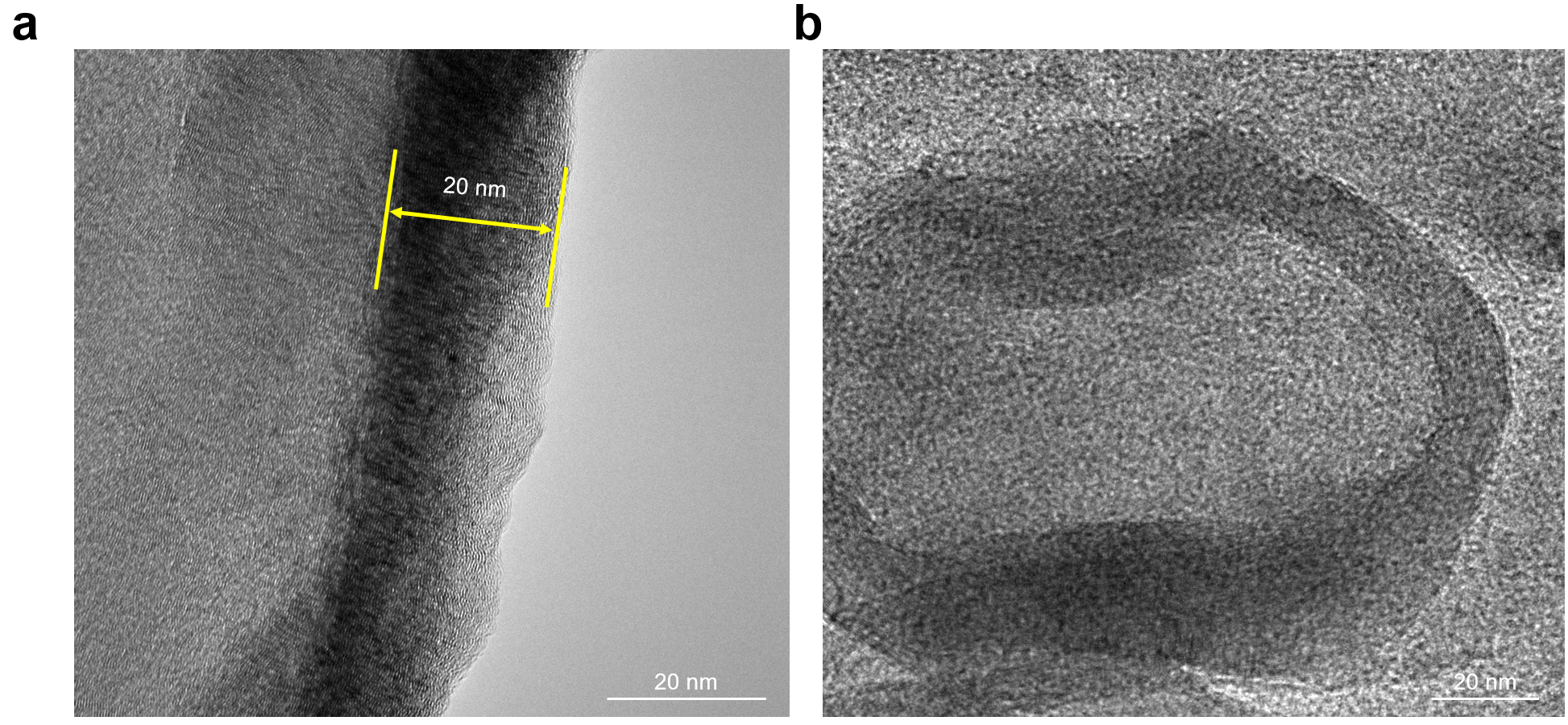


**Figure S4.** High-resolution TEM image of (a) microcage and (b) nanocage for NC/Ni(HS)-10.


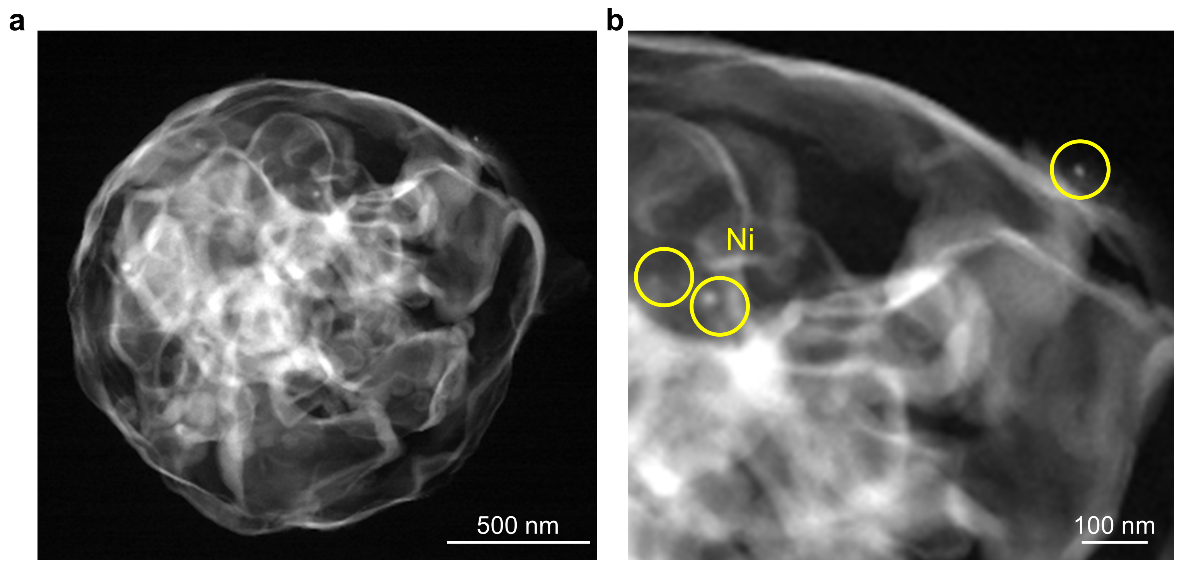


**Figure S5.** HAADF images of NC/Ni(HS).


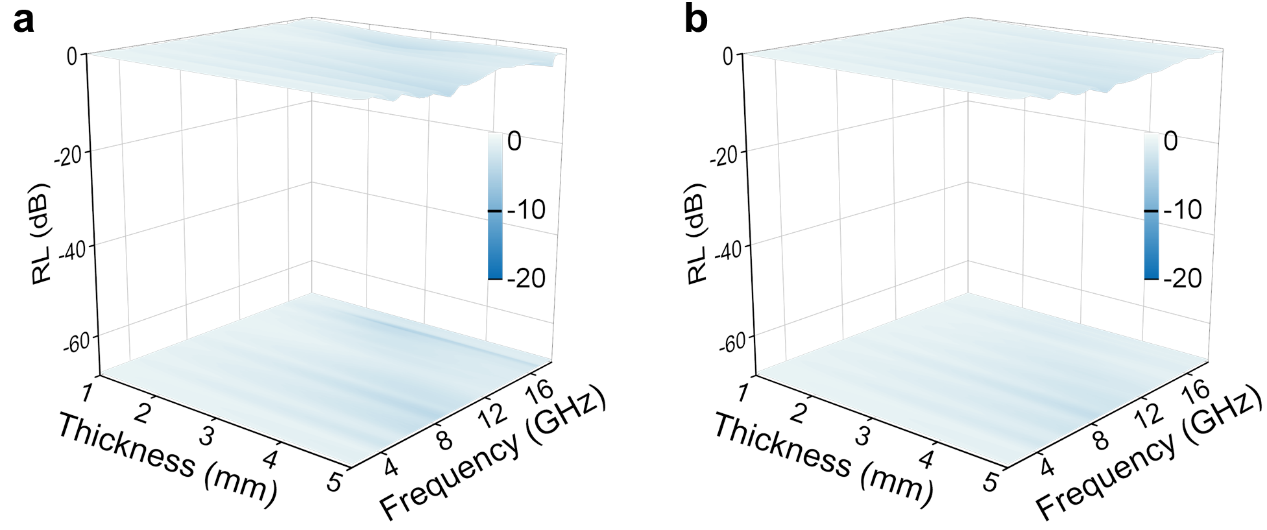


**Figure S6.** 3D RL plots of (a) Ni and (b) NC/Ni.


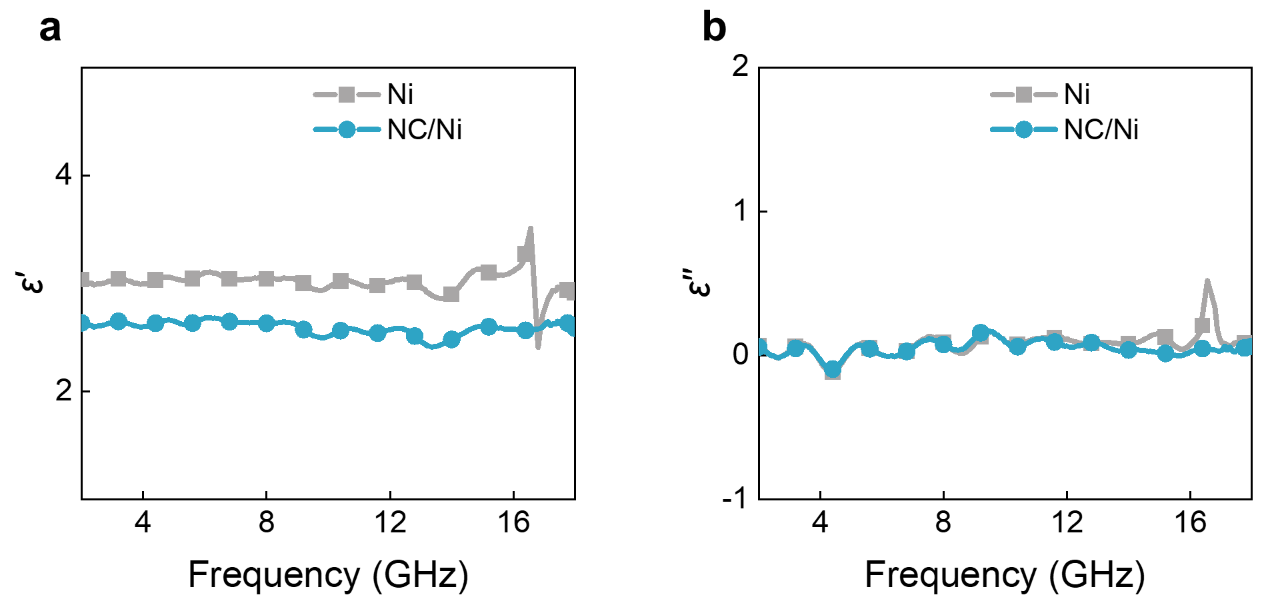


**Figure S7.** (a) *ε*′ and (b) *ε*″of Ni and NC/Ni.


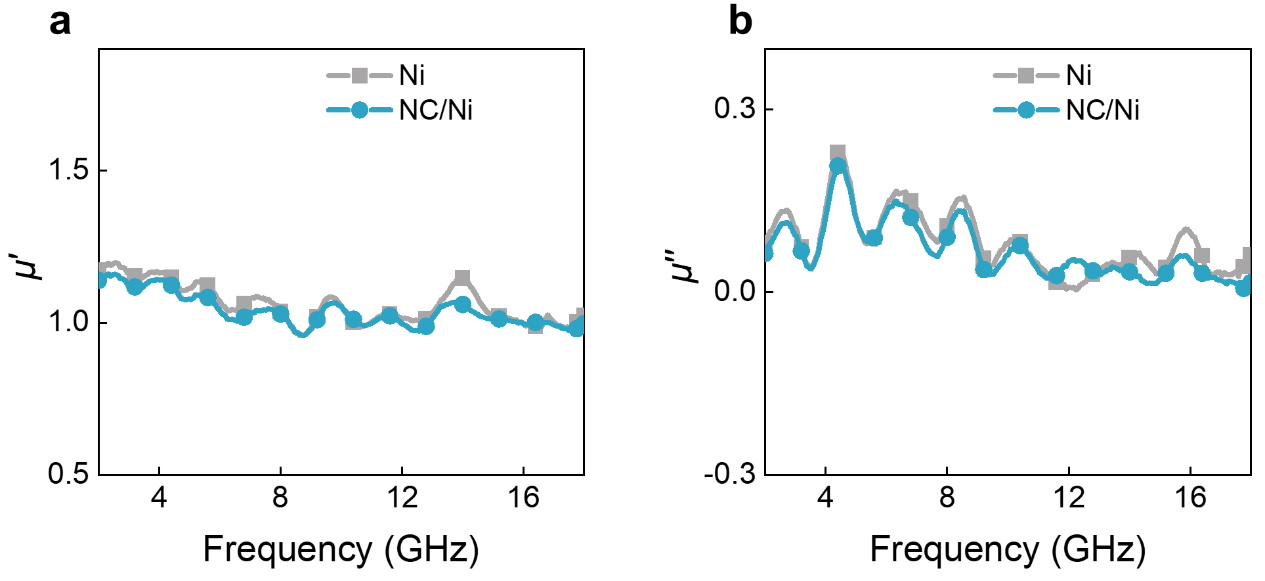


**Figure S8.** (a) *μ*′ and (b) *μ*″ of Ni and NC/Ni.


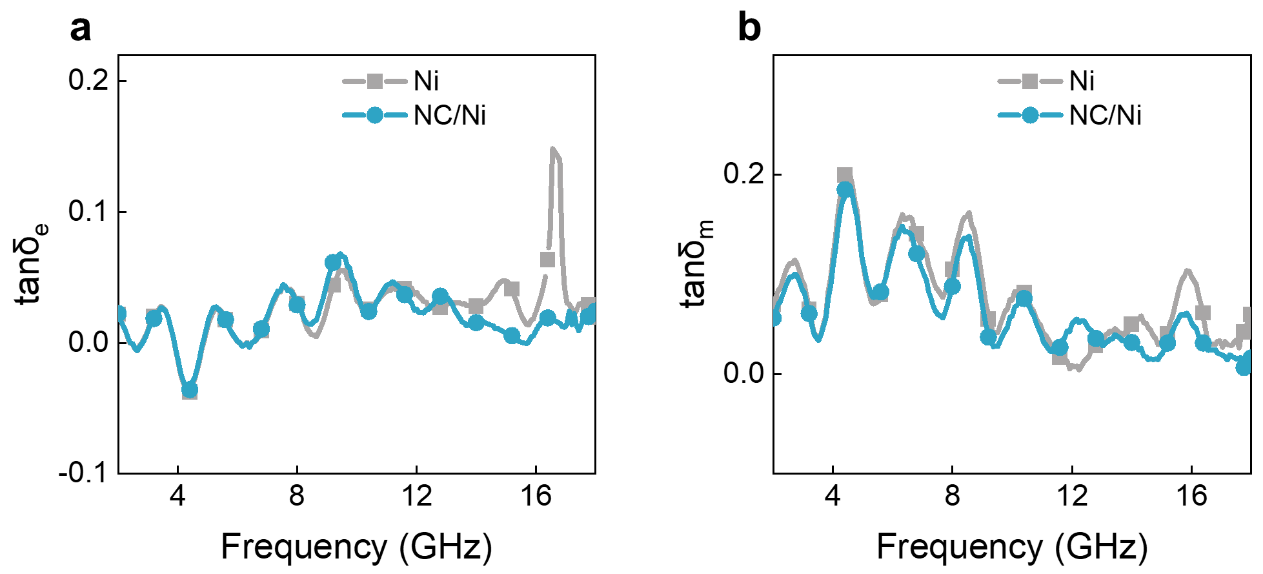


**Figure S9.** (a) tan δ_e_ and (b) tan δ_m_ of Ni and NC/Ni.


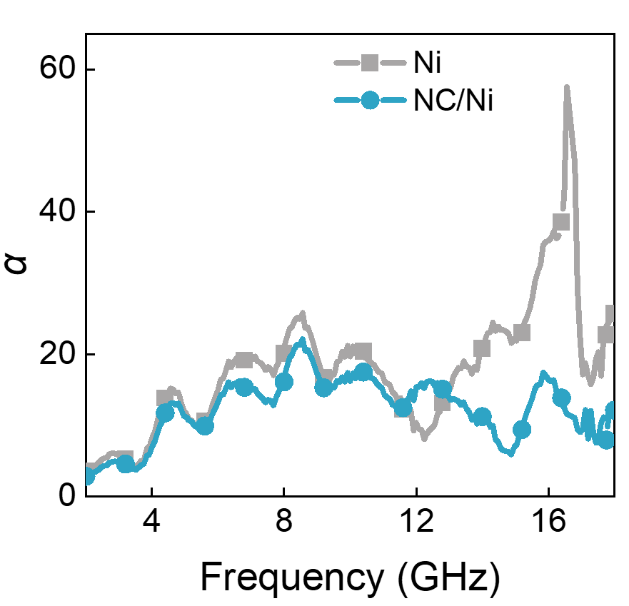


**Figure S10.** Frequency-dependent *α* curves of Ni and NC/Ni.


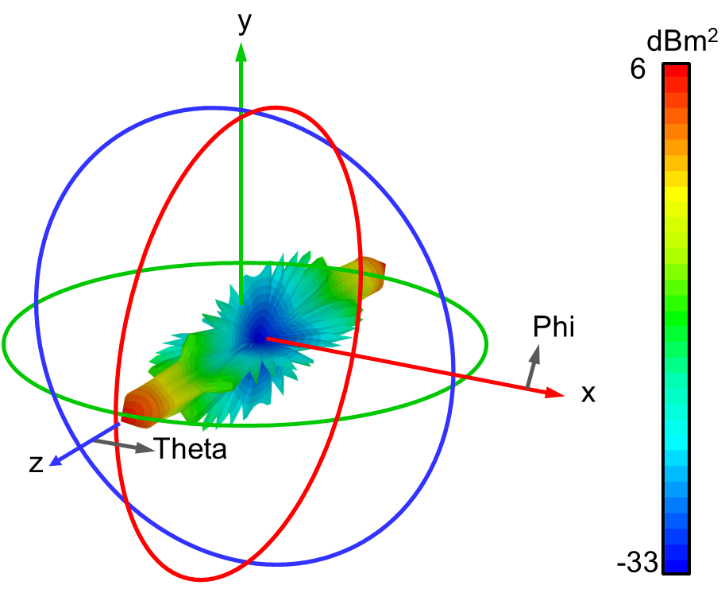


**Figure S11.** 3D RCS diagram for the PEC.
